# CRISPR/Cas9-mediated simultaneous knockout of Dmrt1 and Dmrt3 does not recapitulate the 46,XY gonadal dysgenesis observed in 9p24.3 deletion patients

**DOI:** 10.1016/j.bbrep.2017.01.001

**Published:** 2017-01-09

**Authors:** Masafumi Inui, Moe Tamano, Tomoko Kato, Shuji Takada

**Affiliations:** Department of Systems BioMedicine, National Research Institute for Child Health and Development, Tokyo 157-8535, Japan

**Keywords:** Dmrt1, Dmrt3, CRISPR/Cas9, Gonadal dysgenesis, Sexual development, Testis

## Abstract

DM domain transcription factors play important roles in sexual development in a wide variety of species from invertebrate to humans. Among seven mammalian family members of DM domain transcription factors, DMRT1 has been studied in mouse and human for its conserved role in male gonadal identity. Chromosomal deletion of 9p24.3, the region in which *DMRT1* is located, is associated with 46,XY gonadal dysgenesis. *Dmrt1* knockout (KO) mice also showed male-to-female gonadal reprogramming. However, the phenotype of *Dmrt1* KO mouse appears only after birth while 46,XY gonadal dysgenesis occurs during the developmental phase, and the cause behind this difference remained unknown. We hypothesized that in human the function of other *DMRT* genes clustered with *DMRT1*, namely *DMRT3*, might also be impaired by the chromosomal deletion, which leads to the gonadal dysgenesis phenotype. Thus, simultaneous loss of multiple DM domain genes in mice could have a more severe impact on gonadal development. To address this issue, we generated double KO mice for *Dmrt1* and *Dmrt3* via the CRISPR/Cas9 system. Comparing adult and neonatal testes of single and double KO mice, we found that loss of *Dmrt1* or *Dmrt3*, or both, does not have apparent effect on male gonadal formation during embryonic development. Our study demonstrated that the discrepancy between human with 9p24.3 deletion and *Dmrt1* KO mouse could not be explained by the simultaneous loss of *Dmrt3* gene. CRISPR/Cas9 is a versatile and straightforward approach to elucidate the questions that were otherwise difficult to address with conventional methods.

## Highlights

• We have generated Dmrt1 KO, Dmrt3 KO and Dmrt1/3 DKO mice via the CRISPR/Cas9 system.
• .CRISPR/Cas9-mediated gene KO could generate 3 KO lines via single microinjection.
• Double KO of *Dmrt1* and *Dmrt3* could not recapitulate human 9p24.3 deletion symptom.

## 1 Introduction

DM domain transcription factors are evolutionarily conserved among metazoan species and are involved in the gonadal development in a wide range of species [1], [2], [3], [4], [5]. In mammal (mouse and human), seven *Dsx*- and *mab‐3*-related transcription factor (Dmrt) family genes are identified. Among them, *Dmrt1* has been studied for its pivotal role in maintaining male gonad identity [2], [6], [7] and female germ cell maturation [8], [9]. Indeed, human with chromosomal deletion of 9p24.3, the region in which *DMRT1* is located, are often associated with 46,XY gonadal dysgenesis [10], [11]. In mouse, *Dmrt1* deficiency also caused the degeneration and feminization of male gonad, thus partly recapitulating the etiology of human patients of 9p24.3 deletion. However, the gonadal phenotypes observed in *Dmrt1* KO mice are apparent only after birth. The embryonic development of male gonad is essentially normal in those mice, which shows stark difference from the etiology of human 9p24.3 deletion in which prenatal feminization is observed. Moreover, despite the phenotypic variation among the patients with 9p24.3 deletion, at least some patients with clear 46,XY gonadal dysgenesis phenotype retain normal *DMRT1* exon sequences in their normal chromosome 9, implying haploinsufficiency of the *DMRT1* gene in humans [11], [12]. On the other hand, heterozygous *Dmrt1* mutant mice do not show abnormality [2].

These differences between human and mouse could be due to the distinct roles of DMRT1 in two species, or the other Dmrt family genes with redundant function that are encoded beside the *Dmrt1* gene. In particular, *Dmrt1*, *Dmrt3* and *Dmrt2* genes are clustered within 200 kb regions in both mouse and human genome, and deleted regions observed in 9p24.3 deletion patients also include *DMRT3* and/or *DMRT2* genes [10]. Thus, simultaneous loss of function of DMRT1 and DMRT3 (or DMRT1, DMRT2, and DMRT3) in those patients could have more severe effect on gonadal development than single gene knockout. Accordingly, small deletions that affect only several exons of the *DMRT1* gene or regulatory variants of *DMRT1* are found in patients with milder gonadal dysfunction, such as male infertility [13], [14].

Previous studies have shown that while *Dmrt2* expression is absent in the gonad, *Dmrt3* is expressed in the male gonad in chicken and mouse [15], [16]. Moreover, genetic loss of *Dmrt2* does not cause any gonadal defect [17], but *Dmrt3* deficient mouse is reported to show male sexual developmental abnormality [18]. These studies prompted us to focus on *Dmrt3* rather than *Dmrt2* to examine if loss of another Dmrt gene in addition to *Dmrt1* could cause a more severe phenotype. However, as *Dmrt1* and *Dmrt3* genes are clustered within 100 kb, it is almost impossible to obtain double KO mice by mating single KO mice of two genes. To this end, we decided to utilize the CRISPR/Cas9 system to achieve simultaneous loss of function of *Dmrt1* and *Dmrt3* genes in mouse.

The CRISPR/Cas9 system is a recently developed technique for engineering the genome of the organism *in vivo*, where guide RNAs (gRNAs) recognize target genomic loci by the sequence complementarity and recruit Cas9 nuclease to introduce double strand breaks (DSBs) to the loci [19], [20]. Upon cleavage of genomic DNA, cellular DNA damage response mechanisms repair the DSBs, but small insertions or deletions (indels) could be introduced to the target sites which could cause frame-shift mutations. The CRISPR/Cas9 system could also be applied to mouse mutagenesis via RNA microinjection into the zygotes, and its high efficiency in introducing mutations has been reported [21], [22], [23]. In this study, we took advantage of this system to generate single and simultaneous KO mice of *Dmrt1* and *Dmrt3* genes, and examined their role in male gonad development.

## 2 Material and methods

### 2.1 gRNA synthesis and microinjection

gRNAs were synthesized and microinjected as described previously [21]. Human codon-optimized Cas9 (hCas9) and sgRNA cloning vector a gift from George Church (Addgene plasmid #41815 and #41824, respectively) [20]. Essentially, sequences that recognize *Dmrt1* and *Dmrt3* target sites were introduced into gRNA cloning vector through inverse PCR. gRNA sequences were PCR amplified from the plasmids and served as templates for the *in vitro* transcription by mMessage mMachine T7 kit (Thermo Fisher Scientific, Waltham, MA, USA). Transcribed gRNAs were purified with Megaclear (Thermo Fisher Scientific) and ethanol precipitation and microinjected into the cytoplasm of the fertilized eggs obtained from intercross of F1 hybrid (C57BL/6×DBA/2) BDF1 (Sankyo Labo Service Corporation). The concentrations of the RNAs are as follows: Dmrt1 gRNA, 166 ng/μl; Dmrt3 gRNA, 166 ng/μl; and hCas9 mRNA, 166 ng/μl. Primer sequences used for the cloning and template amplification of the gRNAs are listed in Supplementary table 1. All animal protocols were approved by the Animal Care and Use Committee of the National Research Institute for Child Health and Development, Tokyo, Japan.

### 2.2 Identification of mutant allele

Genomic sequences around the target sites of *Dmrt1* and *Dmrt3* genes were PCR amplified with BIOTAQ DNA Polymerase (Bioline, London, UK) from the genomic DNA extracted from tail tip of the mice. The PCR products were either treated with ExoSAP-IT (Affymetrix, Santa Clara, CA, USA) and served for the direct sequencing or cloned into pCR4-TOPO cloning vector (Thermo Fisher Scientific) and sequenced. The mice were judged as mutated if the peaks of an electropherogram of the direct sequence overlapped, and that the overlap began around the gRNA target sites. The mutation rates were calculated as (Number of mice with mutated allele/Number of mice genotyped). Primer sequences used for the genotyping are listed in Supplementary table 1.

### 2.3 Histological examination

Hematoxylin and eosin (H&E) staining and immunofluorescence were performed as described before [24]. Paraffin embedded tissues were sectioned at 7 µm. At least three individuals from one litter, or two individuals from two independent litters were examined for each genotype, and the representative results are shown. The antibodies used were α-Sox9 (Millipore AB5535, 1:1000) (Millipore, Billerica, USA), and TRA98 (Abcam AB82527, 1:1000) (Abcam, Cambridge, UK).

### 2.4 Statistical analysis

Statistical significances are examined using Student's *t*-test.

## 3 Results

### 3.1 Generation of Dmrt knockout mice

**Fig. 1:**
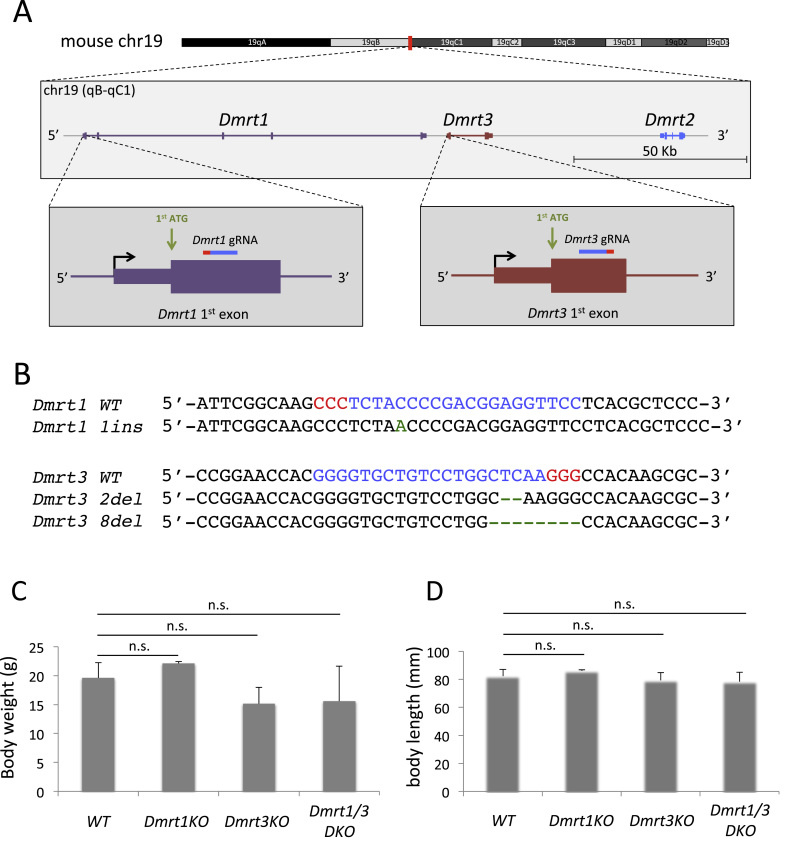
Generation of *Dmrt1KO*, *Dmrt3KO* and *Dmrt1/3DKO* mice. A: A schematic drawing of mouse *Dmrt* gene loci and the positions of gRNAs for *Dmrt1* and *Dmrt3*. Top panel: A schematic drawing of mouse chromosome 19 with colors correspond to G bands. *Dmrt1-2* cluster locus is indicated with the vertical red line. Middle panel: A schematic drawing of the *Dmrt* cluster in chr19 qB-qC1. *Dmrt1*, *3*, and *2* genes are colored in purple, orange and blue, respectively. Introns, UTRs and coding regions are indicated by lines, narrow boxes and wide boxes, respectively. Bottom panel: Schematic drawings around the first exons of *Dmrt1* and *Dmrt3* genes. The positions of Dmrt1 gRNA and Dmrt3 gRNA are indicated with blue bars with red bars indicating PAM sequences. B: Sequence alignments of wild-type (WT) and mutant alleles of *Dmrt1* and *Dmrt3*. The target sequences of Dmrt1 and Dmrt3 gRNAs are indicated with blue letters with red letters indicating PAM sequences. The inserted or deleted bases in mutant sequences are indicated in green letter or green bars, respectively. C: The body weight of WT, *Dmrt1*KO, *Dmrt3*KO, and *Dmrt1/3*DKO mice at 6-week old. Values are mean+SD (n>3). n.s. *P>0.05*. D: The body length of WT, *Dmrt1*KO, *Dmrt3*KO, and *Dmrt1/3*DKO mice at 6-week old. Values are mean+SD (n>3). n.s. *P>0.05.*

gRNAs that recognize the exon 1 of *Dmrt1* and *Dmrt3* genes were designed and cloned into modified gRNA cloning vector (Fig. 1A) [21]. To generate the *Dmrt1* and *Dmrt3* KO mice, the gRNA sequences were PCR amplified, *in vitro* transcribed and microinjected with hCas9 mRNA into the fertilized eggs of BDF1 mouse. The injected embryos were transferred to the pseudo-pregnant female mice and the obtained pups were examined for the genotypes of both *Dmrt1* and *Dmrt3* loci. As a result, we found that at least one allele of *Dmrt1* locus was mutated in 73.9% of the mice genotyped (Table 1). Similarly, 37.3% of the mice examined had indels in *Dmrt3* locus (Table 1). Importantly, 23.2% of the mice had indels in both *Dmrt1* and *Dmrt3* loci, thus those mice potentially had double KO allele (Table 1). However, since it was impossible to discriminate whether the mutations in *Dmrt1* locus and *Dmrt3* locus were on the single chromosome (in *cis*) or on two homologous chromosomes (in *trans*) at this point, we mated the mice with simultaneous mutations with wild-type (WT) C57BL/6 mice and examined the genotype of F1 generation. As a result, we could identify alleles with indels only in *Dmrt1* locus (*Dmrt1*^*1ins*^; *Dmrt3*^*WT*^), *Dmrt3* locus (*Dmrt1*^*WT*^; *Dmrt3*^*8del*^) and both loci (*Dmrt1*^*1ins*^; *Dmrt3*^*2del*^) (Fig. 1B). *Dmrt1*^*1ins*^ allele has 1 base insertion in the exon 1 of *Dmrt1* gene that causes the frame-shift, and the expected protein produced from this allele had premature stop codon and lacked DM domain (Supplementary Fig. S1A). *Dmrt3*^*8del*^ and *Dmrt3*^*2del*^ alleles had 8 and 2 base deletions, respectively, in the exon 1 of *Dmrt3* gene and both mutations caused the frame-shifts. The expected proteins produced from these alleles were also truncated and lacked DM domain (Supplementary Fig. S1B). As the mice with homozygous *Dmrt1*^*1ins*^, *Dmrt3*^*8del*^ or *Dmrt3*^*2del*^ alleles reproduced the previously reported phenotypes of *Dmrt1* and *Dmrt3* KO mice (detailed below), we concluded that we could successfully produce the KO alleles for *Dmrt1* and *Dmrt3* genes. In this manuscript, we designate mice with homozygous *Dmrt1*^*1ins*^; *Dmrt3*^*WT*^, *Dmrt1*^*WT*^; *Dmrt3*^*8del*^, and *Dmrt1*^*1ins*^; *Dmrt3*^*2del*^ allele as *Dmrt1*KO, *Dmrt3*KO, and *Dmrt1/3*DKO, respectively.

**Table 1:** Summary of microinjection targeting the *Dmrt1/3* loci.

Microinjection			Survival of zygotes			Result of genotyping		
hCas9 mRNA	Dmrt1 gRNA	Dmrt3 gRNA	2 cell embryo/injected zygotes	transferred	born	Dmrt1 mutant / genotyped (%)	Dmrt3 mutant / genotyped (%)	Dmrt1 Dmrt3 simultaneous mutant (%)
166 ng/μl	166 ng/μl	166 ng/μl	192/237	192	69	34/46 (73.9)	25/67 (37.3)	16/69 (23.2)

note that some pups were examined only for Dmrt1 or Dmrt3 locus, since we focused to identify double mutant allele.

### 3.2 Analysis of adult testes

*Dmrt1*KO, *Dmrt3*KO and *Dmrt1/3*DKO mice were born in Mendelian ratio without apparent abnormalities (data not shown). However, *Dmrt3*KO and *Dmrt1/3*DKO mice exhibited severe malocclusions as they grew (as reported previously in *Dmrt3* deficient mice [18]). The phenotype is apparent at the weaning, and most of the mice died around the 6th week if untreated, probably due to feeding impairment. Thus, for further analysis we periodically cut their incisors after the weaning to circumvent this problem. In addition, as our purpose was to examine the role of Dmrt genes in male gonad, hereafter we focused only on the male mice. At the 6th week, *Dmrt1*KO, *Dmrt3*KO and *Dmrt1/3*DKO mice had no significant difference in body length or body weight compared with WT mice, except for the slightly lower body weight of *Dmrt3*KO mice (Fig. 1C, D). This could be the primary effect of loss of DMRT3 protein on body growth, or could also be caused by the residual feeding impairment as the slight malocclusions remained even after the treatment.

**Fig. 2:**
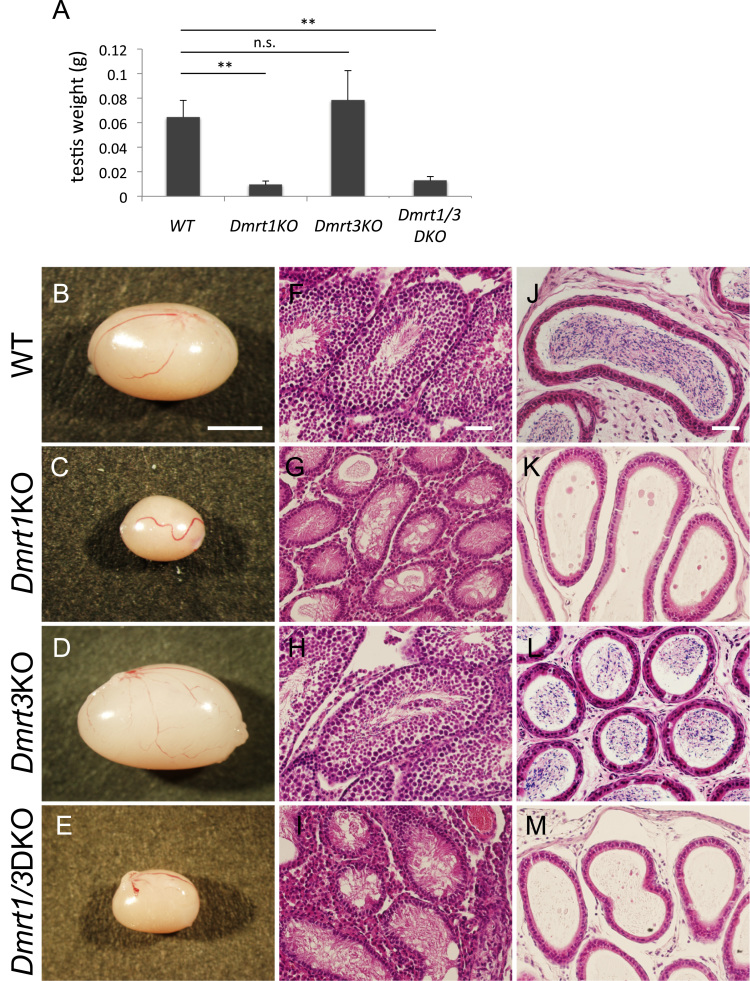
Histological analyses of adult testes. A: The testis weight of WT, *Dmrt1*KO, *Dmrt3*KO, and *Dmrt1/3*DKO mice at 6-week old. Values are mean+SD (n>3). n.s. *P>0.05,* ** *P<0.01*. B-E: Testes of wild-type (WT), *Dmrt1*KO, *Dmrt3*KO, and *Dmrt1/3*DKO mice isolated from 6-week old males. Bar: 2 mm. F–I: H&E staining of the sections of the testes from WT, *Dmrt1*KO, *Dmrt3*KO, and *Dmrt1/3*DKO mice. Bar: 50 µm J-M: Hematoxylin and eosin staining of the sections of the epididymis from WT, *Dmrt1*KO, *Dmrt3*KO, and *Dmrt1/3*DKO mice. Bar: 50 µm.

Next, we focused on the male gonad of these mice. The testes of *Dmrt1*KO mice were smaller and had reduced weight while *Dmrt3KO* testes had no significant difference compared with the WT testes (Fig. 2A–D). The testes of *Dmrt1/3*DKO also showed reduced size and weight, but no significant difference was seen between *Dmrt1*KO and *Dmrt1/3*DKO testes (Fig. 2A, C, E). Histological examination with H&E staining showed essentially normal testes in *Dmrt3*KO mice, with properly formed seminiferous tubules that contained differentiating sperms (Fig. 2F, H). Accordingly, fully differentiated sperms were seen in the epididymis (Fig. 2L). In contrast, testes of *Dmrt1*KO mice were severely degenerated as reported previously (Fig. 2G) [2]. Spermatocyte differentiation was not clear in seminiferous tubules and no mature sperm was observed in the epididymis (Fig. 2G, K). The testes of *Dmrt1/3*DKO mice also showed degeneration of seminiferous tubules and lack of mature sperm in the epididymis (Fig. 2I, M). These results showed that at the 6th week, loss of *Dmrt3* did not affect the male gonad structure or spermatogenesis, and simultaneous loss of *Dmrt1* and *Dmrt3* did not show additive or synergic effects on the testes degeneration caused by *Dmrt1* deficiency.

### 3.3 Analysis of newborn testes

**Fig. 3:**
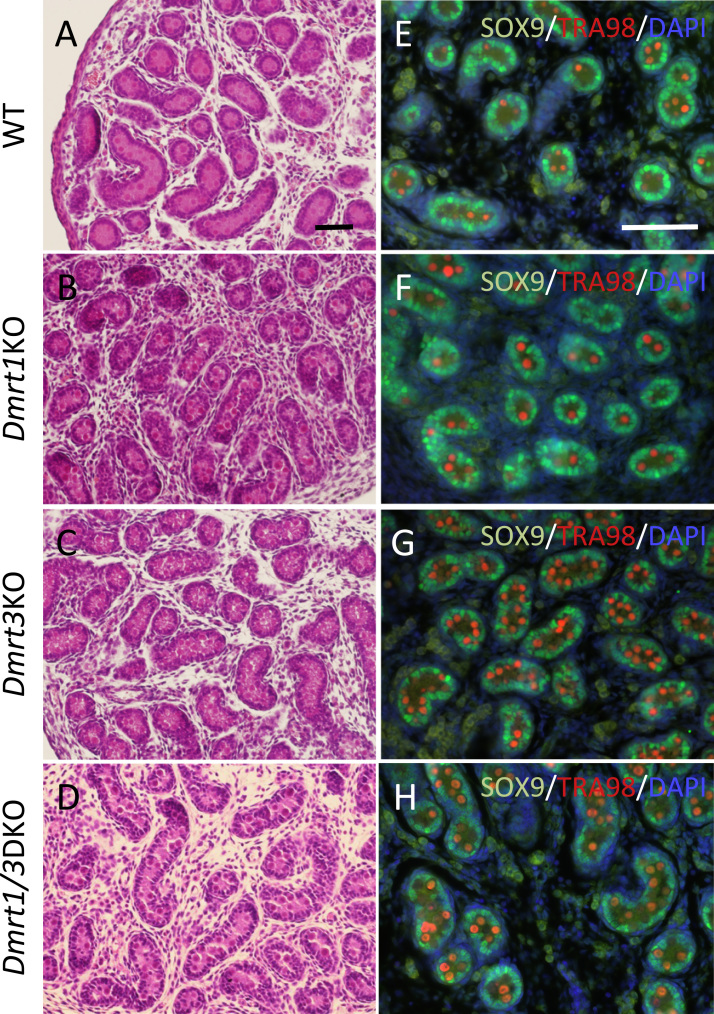
Histological analysis of neonate testis. A-D: Hematoxylin and eosin staining of the sections of the neonate testes from wild-type (WT), *Dmrt1*KO, *Dmrt3*KO, and *Dmrt1/3*DKO mice. Bar: 50 µm. E-H: Immunofluorescence of SOX9 and TRA98 with the neonate testes from WT, *Dmrt1*KO, *Dmrt3*KO, and *Dmrt1/3*DKO mice. Bar: 50 µm.

Given that the adult testes of *Dmrt1/3*DKO were degenerated as those of *Dmrt1*KO, we next examined if loss of both Dmrt proteins had any effect on male gonad developmental process during embryogenesis. Testes of the newborn mice of *Dmrt1*KO, *Dmrt3*KO, *Dmrt1/3*DKO and WT mice were fixed, sectioned and histologically analyzed. H&E staining showed that the testes of *Dmrt1*KO, *Dmrt3*KO and *Dmrt1/3*DKO had no apparent abnormalities compared with those of WT; in all genotypes, seminiferous tubules were formed correctly as WT testes (Fig. 3A-D). Furthermore, immunostaining of SOX9 and TRA98 confirmed that Sertoli cells and germ cells differentiate normally in the testes of the mice with all genotypes (Fig. 3E–H). These results suggested that the loss of DMRT1 or DMRT3, or both, did not cause feminization or apparent developmental deficiency to the male gonad during embryonic development.

## 4 Discussion

In this study, we have generated single and double KO mice for *Dmrt1* and *Dmrt3* genes using the CRISPR/Cas9 system. While the loss of *Dmrt1* gene caused postnatal degeneration of the testes as reported previously [2], no apparent abnormality was observed in the testes lacking *Dmrt3* gene (Fig. 2, Fig. 3). Furthermore, simultaneous loss of *Dmrt1* and *Dmrt3* genes did not cause developmental defect during the embryonic male gonad formation. Our data suggest that *Dmrt3* does not play an indispensable role in male gonad development in mouse, and the defective testicular formation phenotype seen in human patient with 9p24.3 deletion could not be recapitulated by the simultaneous loss of *Dmrt1* and *Dmrt3* genes in mice. Our results indicate that the phenotypic discrepancy between *Dmrt1* KO mouse and 9p24.3 deletion, such as the onset of gonadal abnormality or haploinsufficiency, could not be attributed to the associated loss of *Dmrt3*. The cause of distinct phenotypes in human patient and mouse model remain unclear. This could be due to other genes located around 9p24.3 that might be also impaired, such as *Dmrt2*. Alternatively, this may be due to the distinct role of DM domain proteins in the gonadal development between those two species. DM domain transcription factors are known to play indispensable roles in gonadal development in a wide variety of species, but at the same time, their function in the process changes over the course of evolution [25]. Their role is primary sex determination in some species but is a downstream sex differentiation player in other species. Our result implies the possibility of DM domain transcription factors being different between two mammalian species. Of note, discrepancies of phenotypes between human and mouse with similar genetic modifications are observed also in other reports, for example heterozygous *SOX9* cause XY gonadal malformation in human while testis of *Sox9*^*+/-*^ mouse is histologically normal [26], [27]. Recently, identical p. R92W point mutation in *NR5A1/Nr5a1* gene are reported to cause distinct phenotypes in XX human and mouse [28], [29]. These reports, together with our results, implying that the specific roles of transcription factors during the gonadal development could be distinct between human and mouse.

By microinjecting two gRNAs and hCas9 mRNA into the zygotes, we could successfully generate two single KO and one double KO mice in a single experiment (Table 1). Although we could not directly confirm the loss of DMRT1 and DMRT3 proteins in our KO mice with commercially available antibodies (data not shown), we believe those proteins were depleted from the KO mice, as they exhibited the previously reported phenotypes that are linked with loss of each DMRT proteins; namely, degeneration of adult but not embryonic testes for loss of DMRT1 and severe malocclusion for loss of DMRT3 [2], [18]. High efficiency of the CRISPR/Cas9 system enabled us to produce simultaneous KO of two genes located in the vicinity, which was difficult to achieve with the conventional method. Although simultaneous KO of *Dmrt1* and *Dmrt3* did not show synergic effect, the usefulness of this methodology in answering the questions that were otherwise difficult to address with conventional technique should be stressed.

*Dmrt1* deficiency in gonadal somatic cells causes the up-regulation of female gonad specific genes such as *Foxl2* in adult XY gonad [6], and hence it may be interesting to assess whether loss of *Dmrt3* also causes any feminization of male gonads in the long term. For this purpose, however, gonad-specific KO mouse will be desired to exclude any indirect effect of weight loss caused by feeding impairment. *Dmrt3* deficiency has been reported to cause “male sexual developmental abnormality” [18], but in this study we did not observe an apparent phenotype on sexual development or fertility in *Dmrt3KO* mice. This could be due to the difference in the genetic background or to the methodology of generating KO mice. In addition, severe malocclusion caused by the loss of *Dmrt3* prevents the precise and quantitative evaluation of their potential for the reproduction. Thus, tissue-specific KO would be necessary for further detailed investigation. *Dmrt1* has both Sertoli cell and germ cell autonomous roles during male gonad development [6], [30], [31], [32]: the former is to maintain male identity of supporting cells by preventing the expression of female genes such as *Foxl2*, and the latter is to support the survival and radial migration of male germ cells. Importantly, the role of *Dmrt1* in germ cells was revealed only under cell-type specific knockout, because loss of *Dmrt1* in Sertoli cell indirectly affects germ cells and covers the relatively minor phenotype of loss of *Dmrt1* in germ cells. As our *Dmrt3* KO mice are non-conditional and thus affect both Sertoli and germ cells, it will be interesting to examine also germ cell specific role of *Dmrt3* in future study.

## 5 Conclusion

In conclusion, we have generated single and simultaneous KO mice for *Dmrt1* and *Dmrt3* genes using the CRISPR/Cas9 technique. We found that double knockout of *Dmrt1* and *Dmrt3* genes does not cause sex reversal or developmental deficiency during the embryonic male gonad formation. Our results imply that the discrepancy between the phenotypes of *Dmrt1* KO mice and human with 9p24.3 deletion is not due to the associated impairment of *DMRT3* function, and thus it might depend on the distinct genetic programs for the male gonad formation between the two species.

## Author contributions

T.K. performed the immunofluorescence, M.T. performed the microinjection, and M.I. performed the rest of the experiments. M.I. and S.T. designed the project and wrote the manuscript.

## Competing financial interest

The authors declare no competing financial interests.
