# Supplementary material for: CRISPR/Cas9-mediated simultaneous knockout of Dmrt1 and Dmrt3 does not recapitulate the 46,XY gonadal dysgenesis observed in 9p24.3 deletion patients
Source: Biochem Biophys Rep. 2017 Jan 9;9:238–44. doi: 10.1016/j.bbrep.2017.01.001 (PMC5614593; doi:10.1016/j.bbrep.2017.01.001)
Supplement: Supplementary file 1 — Supplementary material [file mmc1.pdf]

Inui et al. Supplementary Figure 1

A

```
Dmrt1 WT      1 MPNDDTFGKPSTPTEVPHAPGAPPQGKAGGYSKAAGAMAGAAGGSGAGGSGGASGSGPSG   60
Dmrt1 1ins    1 MPNDDTFGKPSNPDGGSSRSRGPAAGQSRRLQQSCRGDGWSGWRLGRGGQRRCLGLRAVG   60
                *********** *         *  *                   * **     *    *

Dmrt1 WT     61 LGSGSKKSPRLPKCARCRNHGYASPLKGHKRFCMWRDCQCKKCSLIAERQRVMAAQVALR  120
Dmrt1 1ins   61 PGFWKQEVPEAAQMRSLQEPRLRVAAQGPQALLHVAGLPVQEVQPDCGATAGDGRAGGPE  120
                 *      *                  *

Dmrt1 WT    121 RQQAQEEELGISHPIPLPSAAELLVKRENNASNPCLMAENSSSAQPPPASTPTPAASEGR  180
Dmrt1 1ins  121 KTAGPGRRTGYQPPNSAAQRSRAPGQKRE                                 149
                         *   *

Dmrt1 WT    181 MVIQDIPAVTSRGHMENTSDLVSDPAYYSSFYQPSLFPYYNNLYNYPQYSMALSAESSSG  240
Dmrt1 1ins  150                                                               149

Dmrt1 WT    241 EVGNSLGGSPVKNSLRSLPAPYVPAQTGNQWQMKTSESRHPVSSQYRMHSYYGPPSYLGQ  300
Dmrt1 1ins  150                                                               149

Dmrt1 WT    301 SMSQIFTFEEGPSYSEAKASVFSPPSSQDSGLVSLSSSSPMSNESSKGVLECESASSEPS  360
Dmrt1 1ins  150                                                               149

Dmrt1 WT    361 SYAVNQVLEEDEDE                                                374
Dmrt1 1ins  150                                                               149
```

B

```
Dmrt3 WT      1 MNGYGSPYLYMGGPVSQPPRAPLQRTPKCARCRNHGVLSWLKGHKRYCRFKDCTCEKCIL   60
Dmrt3 2del    1 MNGYGSPYLYMGGPVSQPPRAPLQRTPKCARCRNHGVLSWQGPQALLSLQGLHLREVHPD   60
Dmrt3 8del    1 MNGYGSPYLYMGGPVSQPPRAPLQRTPKCARCRNHGVLSW--PQALLSLQGLHLREVHPD   58
                ****************************************               *

Dmrt3 WT     61 IIERQRVMAAQVALRRQQANESLESLIPDSLRALPGPPPPGDAAATAATASQSSPASQAS  120
Dmrt3 2del   61 HRAAAGDGGAGGAAQAAGQREPGEPHPGLAAGSARAPAAGGRCGHRRYRLAVVASLPSVP  120
Dmrt3 8del   59 HRAAAGDGGAGGAAQAAGQREPGEPHPGLAAGSARAPAAGGRCGHRRYRLAVVASLPSVP  118
                         *  *       *  *            *   *

Dmrt3 WT    121 QPPAPPRPTAELAAAAALRWVAEPQPGTLPAQLAKPDLTEERVGDSSSTDNTAEAFSDKD  180
Dmrt3 2del  121 AARAASSHRGVGRGRRAALGRGAPARDAASAAC---------------------------  153
Dmrt3 8del  119 AARAASSHRGVGRGRRAALGRGAPARDAASAAC---------------------------  151
                   *            *      *      *

Dmrt3 WT    181 TDQRSSPDVVKSKNCFTPESPEIVSVDEGGYAVQKNGGNPESCPDSPKYHAEQSHLLIEG  240
Dmrt3 2del  154 ------------------------------------------------------------  153
Dmrt3 8del  152 ------------------------------------------------------------  151

Dmrt3 WT    241 PSGTVSLPFSLKANRPPLEVLKKIFPNQKPTVLELILKGCGGDLVSAVEVLLSSRSSAAG  300
Dmrt3 2del  154 ------------------------------------------------------------  153
Dmrt3 8del  152 ------------------------------------------------------------  151

Dmrt3 WT    301 AERTAEESLVLPSSGHIFEHTLGSYPISSSKWSVGSAFRVPDTLRFSADSSNVVPNPLAV  360
Dmrt3 2del  154 ------------------------------------------------------------  153
Dmrt3 8del  152 ------------------------------------------------------------  151

Dmrt3 WT    361 PLQHPFPQPPRYPLMLRNTLARNQSSPFLPNDVTLWNTMTLQQQYQLRSQYVSPFPSNST  420
Dmrt3 2del  154 ------------------------------------------------------------  153
Dmrt3 8del  152 ------------------------------------------------------------  151

Dmrt3 WT    421 SVFRSSPVLSSRTTEDPRISIPDDGCPIVTKQSIYTEDDYDERSDSSDSRILNTSS      476
Dmrt3 2del  154 --------------------------------------------------------      153
Dmrt3 8del  152 --------------------------------------------------------      151
```

Supplementary Figure legends

Supplementary Figure 1

(A) Alignment of deduced amino acid sequences of *Dmrt1* alleles. Top row shows the sequence of wild-type (WT) allele and bottom row shows 1ins allele. Red letters indicate the DM domain. Blue arrow indicates the position of frame shift caused by the insertion and stars indicate the identical amino acids between two sequences. The numbers shown on both sides indicates the number of amino acids. (B) Alignment of deduced amino acid sequences of *Dmrt3* alleles. Top row shows the sequence of WT allele and middle and bottom rows show 2del and 8del alleles, respectively. Red letters indicate the DM domain. Blue arrow indicates the position of frame shift caused by the insertion and stars indicate the identical amino acids between three sequences. The numbers shown on both sides indicate the number of amino acids.

Supplementary Table 1. Name and sequences of the primers

primer name	primer sequences (5'-3')
Dmrt1_gRNA_fw	GAACCTCCGTCGGGGTAGAGTTTTAGAGCTAGAAATAGCAAG
Dmrt1_gRNA_rev	AACTCTACCCCGACGGAGGTTCCGGTGTTTCGTCCTTTCCAC
Dmrt3_gRNA_fw	GGGTGCTGTCCTGGCTCAAGTTTTAGAGCTAGAAATAGCAAG
Dmrt3_gRNA_rev	AACTTGAGCCAGGACAGCACCCCGGTGTTTCGTCCTTTCCAC
Dmrt1_gRNA-T7	TTAATACGACTCACTATAGGGAACCTCCGTCGGGGTAGA
Dmrt3_gRNA-T7	TTAATACGACTCACTATAGGGGGTGCTGTCCTGGCTCAA
Dmrt1_genotyping_fw	CAAGACTCCGGCTTTTCTTG
Dmrt1_genotyping_rev	GGCAGCTTTGCTGTAACCTC
Dmrt3_genotyping_fw	AGGAAGTGGCTCAGACCGTA
Dmrt3_genotyping_rev	TGATCAGGATGCACTTCTCG
